# A novel software tool for semi-automatic quantification of thoracic aorta dilatation on baseline and follow-up computed tomography angiography

**DOI:** 10.1007/s10554-018-1488-9

**Published:** 2018-12-14

**Authors:** Xinpei Gao, Sara Boccalini, Pieter H. Kitslaar, Ricardo P. J. Budde, Shengxian Tu, Boudewijn P. F. Lelieveldt, Jouke Dijkstra, Johan H. C. Reiber

**Affiliations:** 1 Division of Image Processing, Department of Radiology, LUMC, Leiden, The Netherlands; 2 Department of Radiology, University Medical Center, Rotterdam, The Netherlands; 3 LKEB, LUMC, Leiden, The Netherlands; 4 Shanghai Jiao Tong University, Shanghai, China

**Keywords:** Aorta, Thoracic, Computed tomography angiography, Follow-up studies, Dimensional measurement accuracy, Semi-automatic data processing, Segmentation, Software assessment

## Abstract

A dedicated software package that could semi-automatically assess differences in aortic maximal cross-sectional diameters from consecutive CT scans would most likely reduce the post-processing time and effort by the physicians. The aim of this study was to present and assess the quality of a new tool for the semi-automatic quantification of thoracic aorta dilation dimensions. Twenty-nine patients with two CTA scans of the thoracic aorta for which the official clinical report indicated an increase in aortic diameters were included in the study. Aortic maximal cross-sectional diameters of baseline and follow-up studies generated semi-automatically by the software were compared with corresponding manual measurements. The semi-automatic measurements were performed at seven landmarks defined on the baseline scan by two operators. Bias, Bland–Altman plots and intraclass correlation coefficients were calculated between the two methods and, for the semi-automatic software, also between two observers. The average time difference between the two scans of a single patient was 1188 ± 622 days. For the semi-automatic software, in 2 out of 29 patients, manual interaction was necessary; in the remaining 27 patients (93.1%), semi-automatic results were generated, demonstrating excellent intraclass correlation coefficients (all values ≥ 0.91) and small differences, especially for the proximal aortic arch (baseline: 0.19 ± 1.30 mm; follow-up: 0.44 ± 2.21 mm), the mid descending aorta (0.37 ± 1.64 mm; 0.37 ± 2.06 mm), and the diaphragm (0.30 ± 1.14 mm; 0.37 ± 1.80 mm). The inter-observer variability was low with all errors in diameters ≤ 1 mm, and intraclass correlation coefficients all ≥ 0.95. The semi-automatic tool decreased the processing time by 40% (13 vs. 22 min). In this work, a semi-automatic software package that allows the assessment of thoracic aorta diameters from baseline and follow-up CTs (and their differences), was presented, and demonstrated high accuracy and low inter-observer variability.

## Introduction

Aortic aneurysms are the second most frequent disease of the aorta after atherosclerosis. The estimated risk of rupture or dissection depends on the maximal cross-sectional diameter of the aneurysm, which is also the most important parameter to decide if and when to undergo surgery or percutaneous intervention [1, 2]. For patients with aortic dilatation who do not meet the criteria for intervention, imaging follow-up is recommended to monitor diameters at intervals that vary depending also on the underlying aortic pathology. Aortic dilatations/aneurysms are a manifestation of a diffuse aortic pathology and therefore the entire aorta, not only the enlarged segment, should be assessed both at baseline and at follow-up. To reduce variability between institutions and/or operators, measurements of the aorta should be performed at several specific predefined landmarks and reported accordingly [1–3].

CT is the imaging modality of choice to measure aortic diameters. Measurements have to be performed in a plane perpendicular to the long axis of the vessel that can be identified manually or by semi-automatic/automatic software [1, 3]. The manual technique requires a workstation for multiplanar reconstructions, knowledge and experience on how to obtain the correct planes, about the aortic anatomy as well as the positions of specific landmarks. Moreover, for each exam and at all locations, the operator must repeat the process to define the planes perpendicular to the long axis of the aorta ensuing a very time consuming post-processing procedure, especially when the baseline scans have to be reassessed as well. Several commercially available semi-automatic and automatic software packages are available and able to detect the aortic centerline and aortic diameters, reducing the reporting time and measurement variability especially among non-expert readers [4–8].

A single software package that would be able to semi-automatically/automatically calculate the differences in aortic diameters from multiple scans of the same patient, would reduce reporting time further and likely decrease the inter-observer variability. Kauffmann et al. [4, 5] developed a semi-automatic tool to compare volumes and diameters of the abdominal aorta of two successive scans. However, to the best of our knowledge there is no such tool currently available for the thoracic aorta.

Therefore, the aim of this study was to analyze the accuracy and inter-observer variability of our newly developed tool for the semi-automatic assessment of thoracic aorta diameters changes over time by comparison with manual measurements.

## Methods

### Study population and CT protocol

In this single-center retrospective study, for which a waiver for informed consent was received from the local Medical Ethics Committee, two CT scans of patients who had shown an increase of thoracic aorta diameters over time were included. To identify these patients, the PACS of the Erasmus Medical Center was searched for radiological reports of CT scans performed between 2006 and March 2016 including the following predefined terms: “more dilated”, “increase in diameter”, “increased dilatation”, “wider dilatation”, “wider aneurysm”, and “change in diameter”. The 111 patients with reports containing any of these search phrases, were eligible for inclusion regardless of the amount of diameter increase. Next, the quality of the corresponding CT scan and of the one used as a comparison for clinical purposes, was subjectively assessed by a radiologist with 4 years of experience in cardiovascular radiology regarding the presence of motion artifacts and contrast opacification of the aorta. A 4-point scale was employed with the following image quality grades: (1) not acceptable, being non-diagnostic images with not assessable diameters; (2) acceptable, having limited diagnostic value with possible estimation of diameters, but with doubtful reliability at the level of artefacts; (3) good, diagnostic images with limited artefacts allowing a reliable estimation of diameters; and (4) perfect, diagnostic images without artefacts. Only patients who had two contrast enhanced CT scans with qualities judged acceptable to perfect (scale 2–4) were included. Whenever the two so identified scans did not have sufficient quality but the patient had undergone prior and/or later scans that met this criterion, the latter were included. In case multiple exams with adequate qualities were available, the two with the longest time period in-between were selected. The amount and distribution of calcifications and thrombosis were not considered as one of the criteria to classify image quality. All scans that did not have thin slice reconstructions (< 2 mm) with medium-soft convolution kernel of the entire thoracic aorta were excluded. All patients with congenital anatomical variations of the aorta (except for mild aortic coarctation) or who had been operated upon with replacement of any part of the ascending aorta and/or aortic arch prior to the CT scans (except for end-to-end anastomosis for aortic coarctation), were excluded. In total 29 patients whose scans met all the above mentioned parameters were identified.

Patient demographics were retrieved from the electronic patient files. Technical parameters of the CT scans including date, scanner, ECG gating, kV, mAs, reconstruction slice thickness and kernel were collected. The phase of the cardiac cycle at the level of the aortic valve (approximated at 5% intervals) of the reconstruction employed for manual and semi-automatic measurements was noted and, whenever possible, the same phase was chosen to assess the two scans of a single patient.

### Assessment of semi-automatic aorta dilatation quantification software package

The quality of the new software package was assessed by comparing semi-automatically obtained maximal cross-sectional diameter measurements against a manual reference standard. The comparison of semi-automatic and manual diameter values was performed for both baseline and follow-up scans.

### Reference standard

Manual measurements were performed by a radiologist with 4 years of experience in cardiovascular imaging (observer 1) in the use of a multimodality workstation (Syngo.via, Siemens). Measurements were performed on planes perpendicular to the centerline of the aorta that were manually identified with the double-oblique method. Inner-edge to inner-edge maximal cross-sectional diameters were manually defined.

All older scans were assessed first. The more recent scans were assessed at least 2 weeks after the first ones, by the same radiologist blinded to the results of the first datasets. The time needed to perform all the measurements on one dataset was recorded.

### Measurements locations

**Fig. 1:**
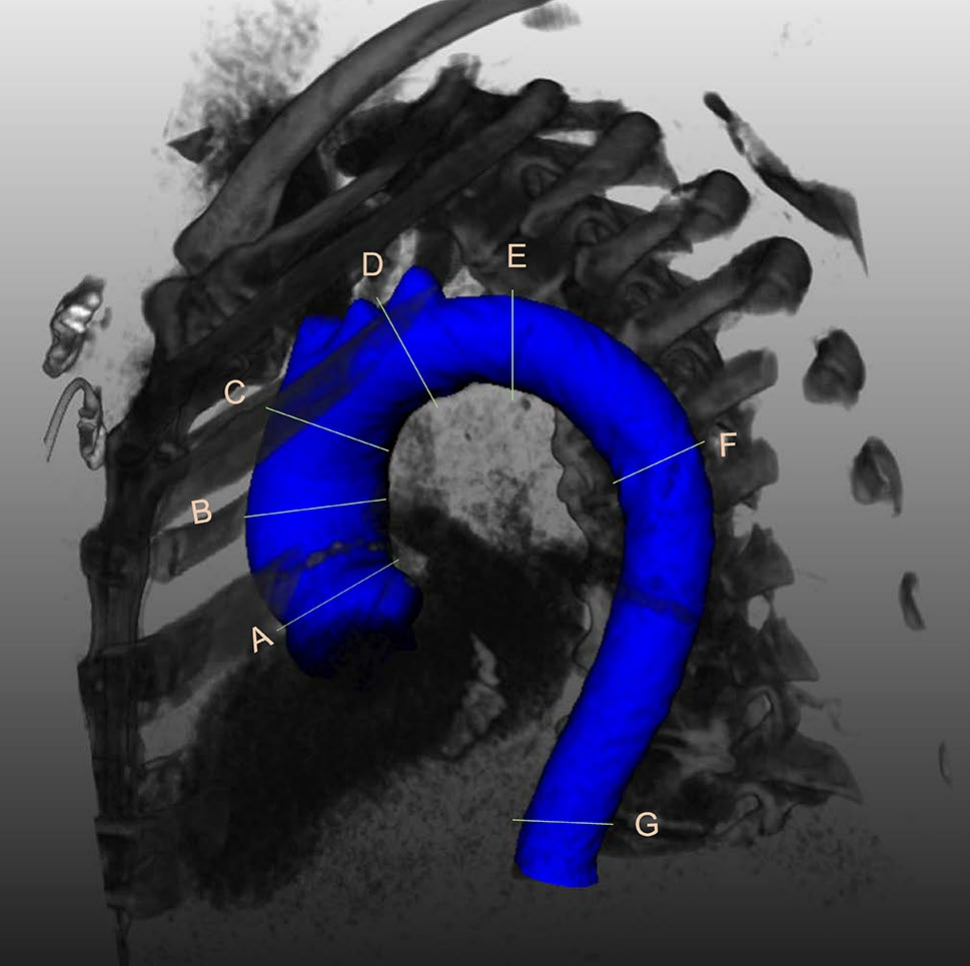
3D reconstruction of the thoracic aorta showing the level of the 7 locations where measurements were performed. A = sinotubular junction (at the connection of the aortic root and the ascending aorta); B = mid ascending aorta (at the level of the pulmonary trunk); C = proximal aortic arch (at the origin of the brachiocephalic trunk); D = mid aortic arch (between the left carotid artery and the left subclavian artery; after the left vertebral artery if it had a separate origin from the aorta); E = proximal descending thoracic aorta (at approximately 2 cm distal to the left subclavian artery; however if at this level there was either a dilatation or a steep bending of the aorta, the plane was moved closer to the left subclavian artery); F = mid descending aorta (at the same level as the MAA); G = diaphragm

To assess changes of aortic dimensions over time, diameters were measured at seven prescribed and standardized anatomical locations in accordance with the 2014 guidelines of the European Society of Cardiology [1] being: sinotubular junction (STJ), mid ascending aorta (MAA), proximal aortic arch (PROX), mid aortic arch, proximal descending thoracic aorta (DIST), mid descending aorta (DESC) and diaphragm. The specifics for each location are described in Fig. 1.

The same landmarks and locations were employed for both manual and semi-automatic measurements.

### Semi-automatic aorta dilatation quantification software

#### Software package overview

**Fig. 2:**
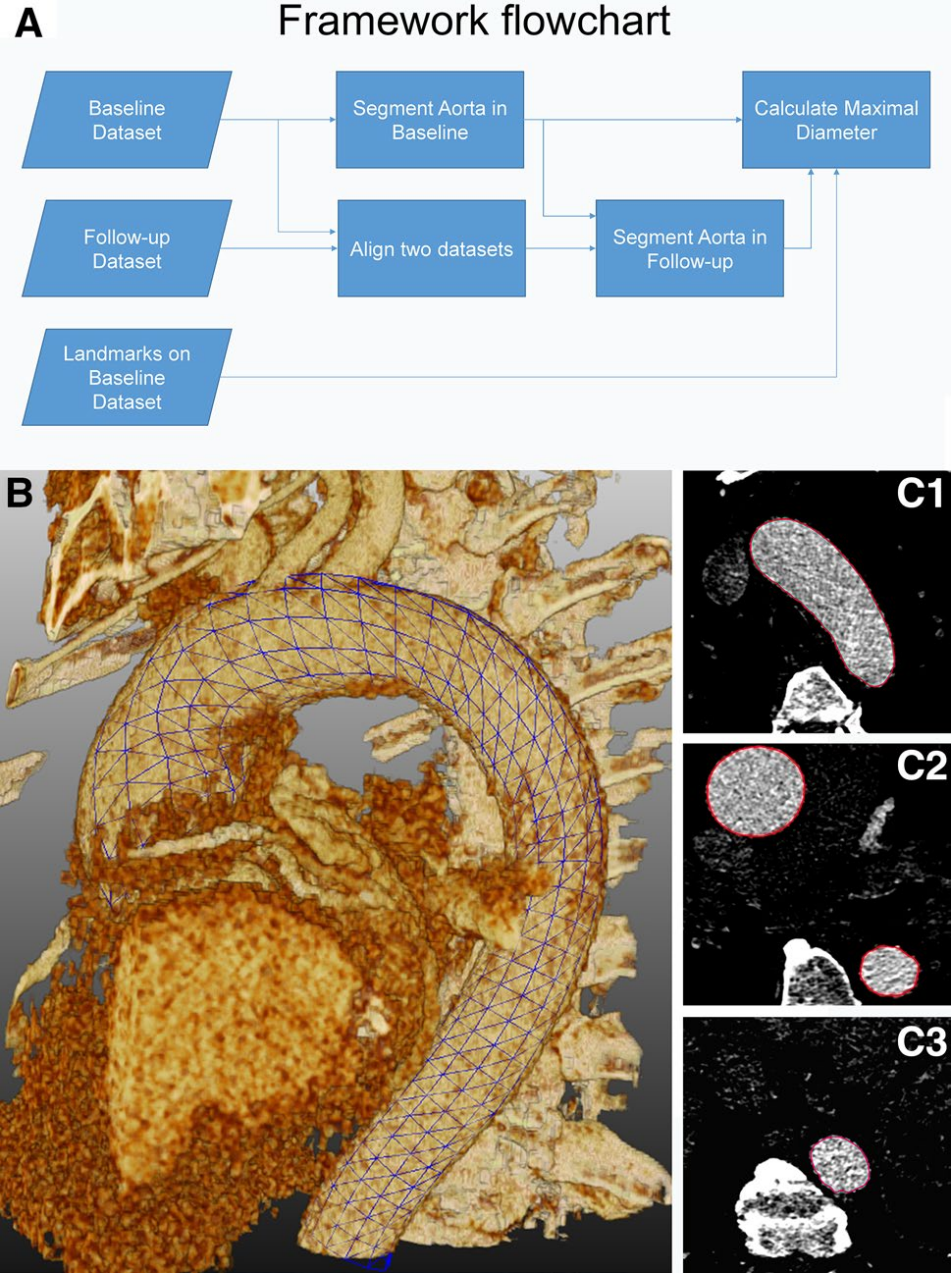
In **a** flowchart representing the main steps that were automated in the software to obtain the measurements from both baseline and follow-up datasets. 3D grid (**b**, blue grid) and 2D axial (**C1–C3**, red contours) views showing the result of semi-automatic segmentation. (Color figure online)

The entire software procedure was constituted of multiple steps, as summarized in Fig. 2a. The inputs were represented by baseline dataset, follow-up dataset and landmarks identified on the baseline dataset. At first, the thoracic aorta was semi-automatically segmented from the baseline CTA images; next, the two datasets (baseline and follow-up) were aligned using the intensity-based registration algorithm. Subsequently, the aorta in the follow-up dataset was segmented. With the segmented contour of the baseline CTA scan as the initial contour, the contour of the thoracic aorta in the follow-up dataset was extracted by deforming the initial contour. Finally, based on the manually defined landmarks on the baseline dataset, the maximal cross-sectional diameters of different locations in baseline and follow-up images were calculated.

The semi-automatic software was implemented in the MeVisLab platform (version 2.7.1, MeVis Medical Solutions AG, Bremen, Germany) using C++ and Python code, and integrated in an in-house tool.

#### Preprocessing

If the length of a scan was longer than the region of the thoracic aorta, for instance extended into the femoral arteries, or if the baseline and follow-up scans had a different length of the aorta that was imaged, the datasets were manually adjusted by removing unnecessary images along the z-axis to obtain two matching datasets of the sole thoracic aorta.

#### Automatic segmentation of baseline CTA

The automatic thoracic aorta segmentation scheme was based on the centerline extraction and subsequent contour detection methods. The centerline extraction method was similar to the algorithm that we developed and described previously [9], based on the wave-propagation algorithm, Gaussian probabilistic distribution model and Dijkstra shortest path algorithm. The previous automatic landmark detection algorithm was modified from the previous algorithm which allows the detection of two femoral end points to the aortic root end point. The contour detection method was implemented as a deformable subdivision surface model fitting algorithm [10]. The result on one particular image is shown in Fig. 2b, c.

#### Automatic alignment of baseline and follow-up CTA

The automatic alignment was implemented by an intensity-based registration (voxel-based registration) algorithm. First, the follow-up image was coarsely aligned to the baseline image by rigid registration. Next, affine registration was implemented for further refinement. By means of this registration, the follow-up image (the moving image) was deformed to fit the baseline image (the fixed image) to find an optimal coordinate transformation. The optimal transformation was obtained when the bias of the baseline and follow-up images reaches minimum value. The bias was evaluated by mutual information, which is the relation between the probability distributions of the intensities. To maximize the mutual information, a stochastic gradient descent algorithm was used to converge to the optimal value. To avoid the influence of structures such as the rib cage, a mask including the aorta was generated by minimum bounding box of the segmented thoracic aorta in the baseline CTA. The mask was used as the region of interest for the fixed image during registration.

In our study, the Elastix open source toolbox [11] was used for the intensity-based registration.

#### Automatic segmentation of follow-up CTA

The aligned follow-up CTA image was processed by the centerline-based adaptive threshold method [12] to reduce the influence of the surrounding tissues in the background, such as high intensity tissue like bone, and low intensity tissue like muscle.

With the alignment of the baseline and follow-up images as described in the previous step, the position and shape of the aorta in the two images became the same with the exception of differences due to the dilatation. By using the aligned follow-up CTA image as the cost function image and the baseline aorta contour as the initial contour, the subdivision surface fitting algorithm will deform the initial contour to detect the edge with the highest gradient in the cost function image [12]. Following the segmentation of the aorta by subdivision fitting, a region-growing algorithm was used to detect in detail the aortic arch and its branches.

#### Manual definition of landmarks for diameter assessment

With the in-house tool, the user could manually annotate the positions of the seven locations where measurements should be performed in multiplanar reconstructions of the baseline scan of each patient. Thereafter, the cross-sectional contours of the aorta could be detected by intersecting the landmark plane with the 3D contour, and the maximum cross-sectional diameter was calculated automatically. The software automatically identified the same locations and derived the aortic diameters at those levels, as well as the differences in diameter compared to baseline, on the follow-up scan.

#### Visualization of diameter progression

**Fig. 3:**
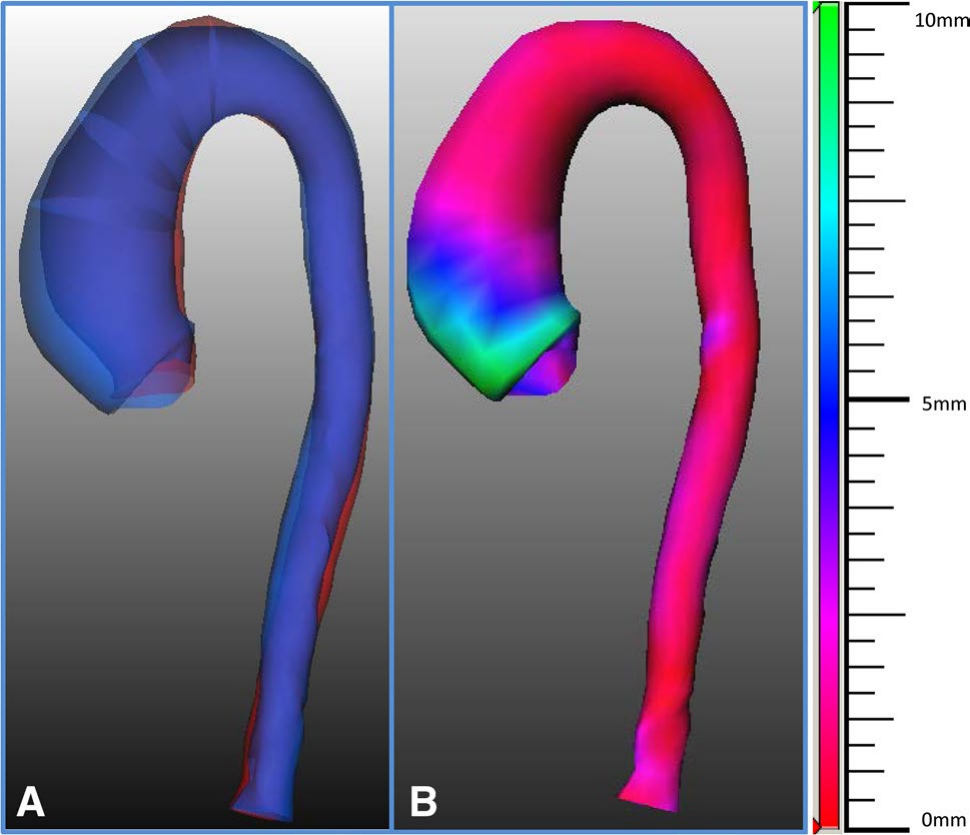
Tools for the visualization of the size changes in the aorta. In **a** superimposed three dimensional views of the surfaces of the thoracic aorta based on the semi-automatically segmented contour of both baseline (in red) and follow-up (in blue) CTA images. In **b** the semi-automatically calculated changes in diameters between the baseline and follow-up scans are represented with colors (red, blue and green indicate 0, 5 and 10 mm differences in diameters, respectively) for an immediate and comprehensive overview of the results. (Color figure online)

**Fig. 4:**
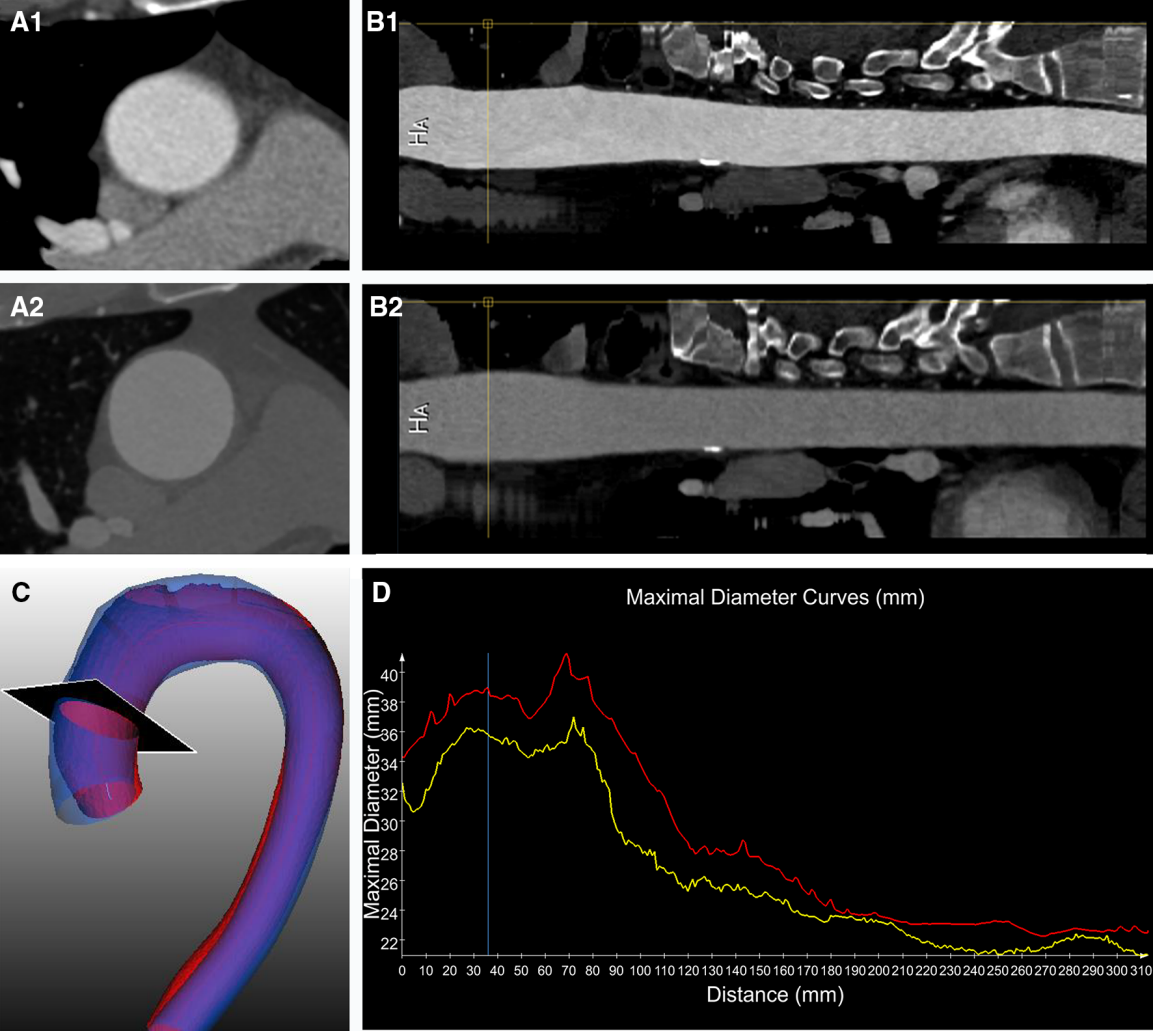
Tools for the visualization of the size change of the aorta. Cross-sectional views of the aorta (**A1** and **A2**) and straightened MPR reconstructions (**B1** and **B2**) of the aligned baseline (**A1** and **B1**) and follow-up (**A2** and **B2**) images. In **C** superimposed 3-dimensional views of the surfaces of the thoracic aorta based on the semi-automatically segmented contours of both baseline (in red) and follow-up (in blue) CTA images. In **D** the two diameters curves (baseline in yellow and follow-up in red), starting from the sinotubular junction, drawn together on the same graph. The black plane in **C** shows the level where the cross-sectional diameter indicated by the yellow lines in **B1** and **B2** and by the blue line in **D** was calculated. (Color figure online)

To improve the visualization of the size changes in the aorta, several graphic presentations were implemented in the software and are illustrated in Figs. 3 and 4.

#### Inter-observer variability

To analyze the inter-observer variability of the in-house tool, two observers (observer 1 and 2) independently used the in-house tool to annotate the seven aortic landmarks manually on the baseline scan of each patient. Observer 2 had 4 years of experience in cardiovascular imaging.

#### Statistical analysis

Statistical analyses were performed using SPSS (version 20.0, SPSS Inc., Chicago, IL, USA) and MedCalc (version 15.6, Ostend, Belgium). Quantitative data was described by the mean, standard deviation, and intraclass correlation coefficient (ICC). To visualize the bias between the semi-automatic results and the reference standard, Bland–Altman plots were created.

## Results

### Patient population

Twenty-nine patients who had two contrast enhanced CT scans with reasonable to perfect quality of the thoracic aorta were included (23 males; average age: 55.5 ± 14.3 years). The patient characteristics are summarized in Table 1.

**Table 1:** Patient population characteristics

	Total (n = 29)
Male	23 (79%)
Age (mean, SD, range) in years	55.5 ± 14.3 (25–82)
Height (mean, SD, range) in m	1.75 ± 0.1 (1.5–1.9)
Weight (mean, SD, range) in Kg	82.6 ± 18 (50–117)
BMI (mean, SD, range)	27.2 ± 6.9 (16.9–52.7)
Congenital aortic valve/aortic diseases	
Ehlers–Danlos type 4	2
Bicuspid aortic valve	5
Coarctation	2
Risk factors for cardiovascular diseases	
Smoking	6 (3 past smokers)
Hypertension	12
Diabetes	4
Hypercholesterolemia	8
Previous related surgical procedures	
Aortic valve replacement	6
Coarctation repair	1
Ross procedure	1

### CT scan technical parameters

In total 58 scans were included, 29 baseline and 29 follow-up examinations. The technical parameters are summarized in Table 2. All but one scans were acquired with scanners with more than 64 detectors. ECG gating or triggering was employed in most of the cases (52 scans; = 90%). The slice thickness of the reconstructions was on average 1 ± 0.2 mm and only in two baseline exams the thickness was bigger than 1 mm.

**Table 2:** CT scans technical parameters

	Baseline CT scans (n = 29)	Follow-up CT scans (n = 29)	Total CT scans (n = 58)
Patient age at CT (average ± SD; range) (years)	50.1 ±13.7; 22–71	53.4 ±14; 24–78	51.7 ± 13.8
Time difference between CT scans (average ± SD; range) (days)		1187.9 ± 622.4; 344–2558	
Scanner			
Sensation 16	1	0	1
Definition	3	0	3
Definition AS+	8	2	10
Definition Flash	12	10	22
Definition Edge	1	1	2
Sensation 64	2	1	3
Somatom Force	2	15	17
kV			
70	1	1	2
80	2	6	8
90	1	5	6
100	12	12	24
110	0	2	2
120	13	3	16
Slice thickness (average ± SD; range) (mm)	1 ± 0.2; 0.75–2	0.97 ± 0.1; 0.75–1	1 ± 0.2
0.75	2	3	5
1	25	26	51
1.5	1	0	1
2	1	0	1
Kernel			
B20f	9	2	11
B25f	1	0	1
B26f	10	1	11
Bv40	2	15	17
I26f	6	11	17
ECG-gating			
Not gated	6	0	6
Unknown protocol	1	2	3
Retrospective	2	1	3
Prospective	7	5	12
Prospective high-pitch	12	21	33
Phase of the cardiac cycle (%)			
0–20	2	4	6
25–40	5	12	17
45–60	5	9	14
65–80	11	3	14

### Accuracy of the tool

From the total of 29 patients included in the study, in only two patients, the automated extraction was not successful. In 1 patient, the extracted centerline was found outside of the vessel; in another patient, the contour was at least 2 mm biased from the boundary of the aorta. These two patients were excluded for further statistical analysis. In the remaining 27 patients (93.1%), no manual interaction was needed for possible modification of the centerline or contours; all the results were compared with the manual measurements.

Table 3 presents the mean and standard deviation of the maximal cross-sectional diameters at different locations obtained by the manual measurement and the semi-automatic assessments by the two observers.

**Table 3:** Average diameter in different locations along the aorta

Maximal diameter (mm)	Baseline			Follow-up		
	Manual	Semi-Automatic 1	Semi-Automatic 2	Manual	Semi-Automatic 1	Semi-Automatic 2
Sinotubular junction	37 ± 5	40 ± 5	39 ± 5	39 ± 5	42 ± 5	42 ± 4
Mid ascending aorta	44 ± 5	43 ± 6	44 ± 6	47 ± 6	45 ± 6	46 ± 6
Proximal aortic arch	37 ± 5	37 ± 4	38 ± 5	39 ± 4	39 ± 4	39 ± 4
Mid aortic arch	29 ± 3	30 ± 5	30 ± 5	30 ± 4	33 ± 5	32 ± 5
Proximal descending aorta	27 ± 5	27 ± 5	27 ± 5	29 ± 7	30 ± 6	29 ± 6
Mid descending aorta	26 ± 4	26 ± 4	26 ± 4	27 ± 4	28 ± 5	27 ± 5
Diaphragm	24 ± 4	24 ± 5	24 ± 4	25 ± 4	25 ± 5	25 ± 5

Manual = measurements performed manually with the double oblique method by observer 1

Semi-automatic 1 and Semi-automatic 2 = semi-automatically calculated diameters based on the locations identified on the baseline scan by observers 1 and 2, respectively. Data are represented as mean ± SD

For observer 1 the mean differences between the manual measurement and the semi-automatic measurement at different landmarks were all less than 1 mm, except at the mid aortic arch, the MAA and the STJ (Table 4). In the baseline and the follow-up scans, the ICC between manual and semi-automatic measurements with landmarks defined by observer 1 were all higher than 0.90.

**Table 4:** Assessment of semi-automatic software compared with manual results

	Sinotubular junction	Mid ascending aorta	Proximal aortic arch	Mid aortic arch	Proximal descending aorta	Mid descending aorta	Diaphragm
Maximal diameter: Semi-Automatic 1 vs. Manual							
Baseline							
Mean difference ±SD (mm)	2.2 ± 2.2	1.1 ± 2.2	0.2 ± 1.3	1.1 ± 2.3	0.2 ± 1.0	0.4 ± 1.6	0.3 ± 1.1
ICC	0.95	0.96	0.98	0.91	0.99	0.95	0.98
Follow-up							
Mean difference ±SD (mm)	3 ± 2.6	1.3 ± 2.5	0.44 ± 2.2	2.4 ± 2.6	0.9 ± 2.8	0.4 ± 2.1	0.4 ± 1.8
ICC	0.92	0.96	0.93	0.90	0.95	0.94	0.96
Maximal diameter: Semi-Automatic 2 vs. Manual							
Baseline							
Mean difference ±SD (mm)	2.0 ± 1.8	0.4 ± 1.7	0.6 ± 1.2	1.2 ± 1.9	0.0 ± 1.4	0.4 ± 2.0	0.4 ± 1.1
ICC	0.97	0.97	0.98	0.94	0.98	0.93	0.98
Follow-up							
Mean difference ±SD (mm)	3.2 ± 2.6	0.3 ± 2.0	0.9 ± 2.1	2.3 ± 2.6	0.6 ± 2.6	0 ± 2.3	0.3 ± 1.6
ICC	0.92	0.97	0.94	0.91	0.96	0.93	0.97

The semi-automatic results were generated based on landmarks defined by observer 1 (semi-automatic 1) and observer 2 (semi-automatic 2)

Also for observer 2 the mean differences at different landmarks were all lower than 1 mm, except at the mid aortic arch and STJ (Table 4). The ICC coefficients for baseline diameters were all higher than 0.90.

**Fig. 5:**
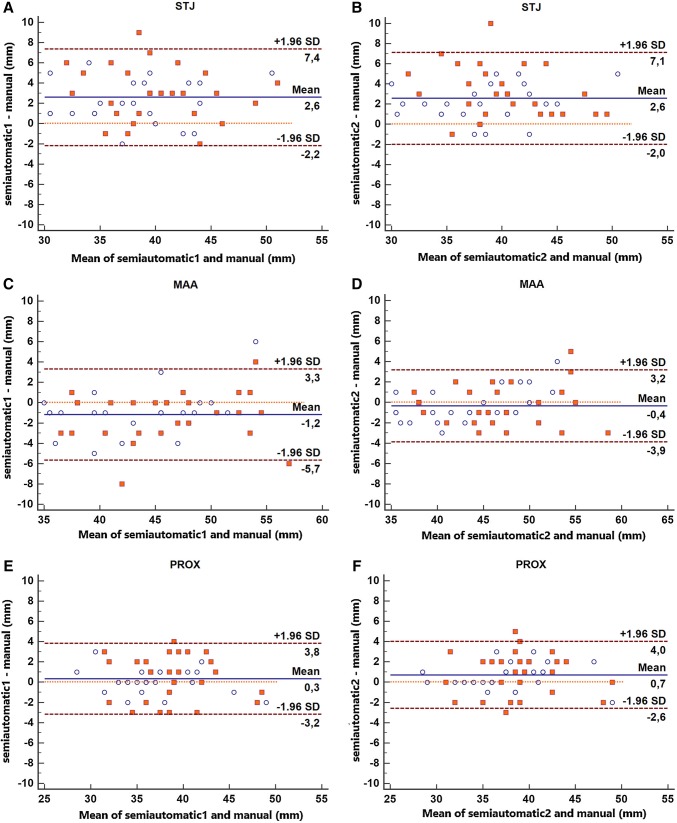
Bland–Altman plots representing the difference between semi-automatic and manual measurements at the sinotubular junction (**a, b**), MAA (**c, d**) and PROX (**e, f**). Blue circles: baseline diameters. Red squares: follow-up diameters. Semi-automatic 1 and Semi-automatic 2 = semi-automatically calculated diameters by observers 1 and 2, respectively. (Color figure online)

**Fig. 6:**
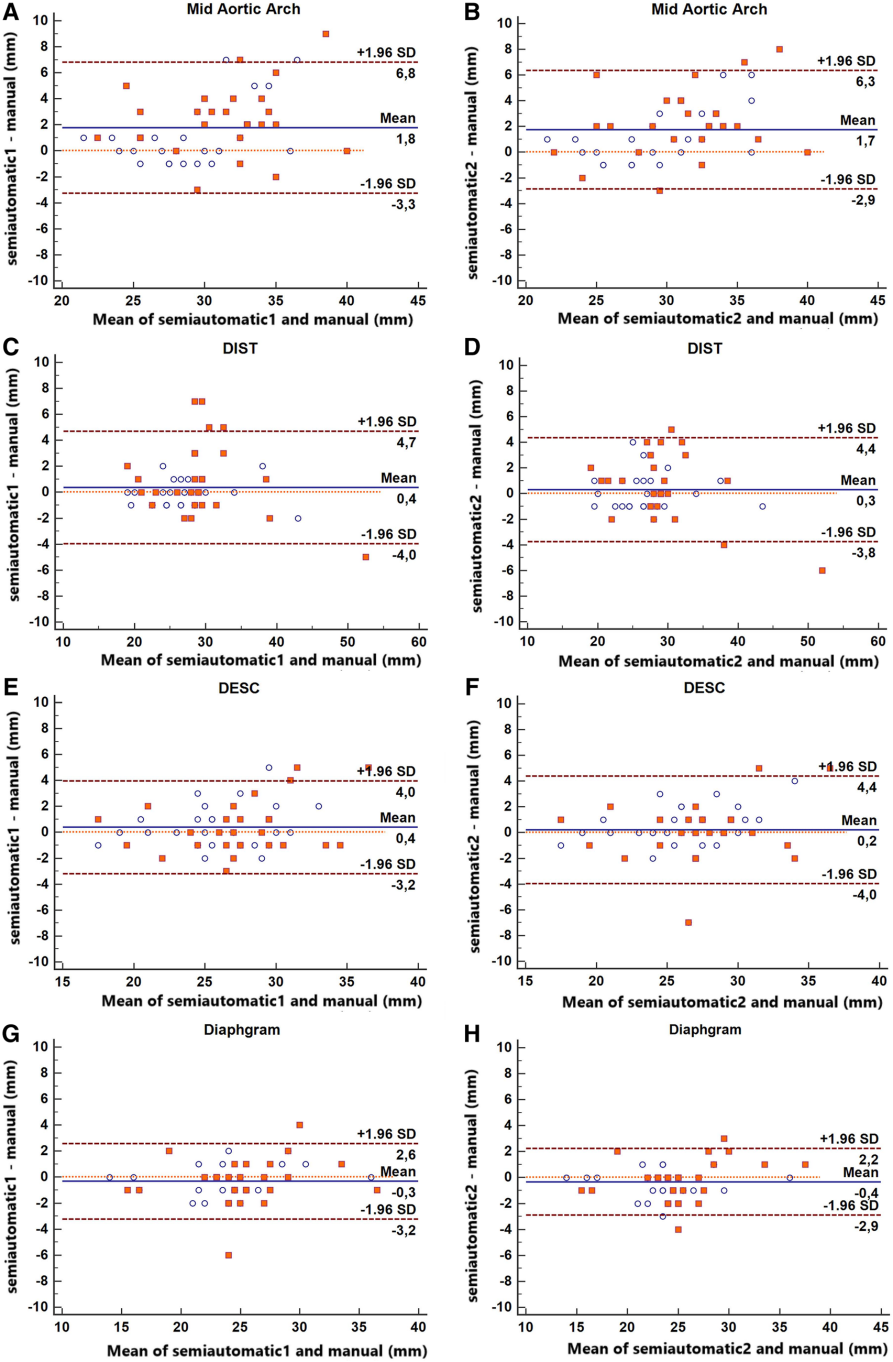
Bland–Altman plots representing the difference between the semi-automatic and manual measurements at mid aortic arch (**a, b**), DIST (**c, d**), DESC (**e, f**) and diaphragm (**g, h**). Blue circles: baseline diameters. Red squares: follow-up diameters. Semi-automatic 1 and Semi-automatic 2 = semi-automatically calculated diameters by observers 1 and 2, respectively

Bland–Altman plots for the differences between the two observers and the semi-automatic results combining data from the baseline and follow-up scans for each of the seven locations are presented in Figs. 5 and 6.

### Inter-observer variability

The semi-automatic software demonstrated a low inter-observer variability (Table 5). The mean differences were around 1 mm at all the locations; the ICC values all higher than 0.90.

**Table 5:** Inter-observer variability for the semi-automatic software

Maximal diameter Semi-Automatic 1 vs. Semi-Automatic 2	Sinotubular junction	Mid ascending aorta	Proximal aortic arch	Mid aortic arch	Proximal descending aorta	Mid descending aorta	Diaphragm
Baseline							
Mean difference ±SD (mm)	0.3 ± 1.4	0.6 ± 2.4	0.4 ± 1.2	0.0 ± 2.0	0.2 ± 1.3	0.0 ± 1.6	0.1 ± 0.8
ICC	0.98	0.96	0.98	0.95	0.99	0.96	0.99
Follow-up							
Mean difference ± SD (mm)	0.2 ± 1.6	1.0 ± 1.9	0.4 ± 1.2	0.1 ± 1.2	0.3 ± 1.4	0.3 ± 1.5	0.1 ± 0.9
ICC	0.97	0.98	0.98	0.98	0.99	0.97	0.99

### Analysis time

The overall mean time needed to manually measure all the diameters on both datasets was 22 min (11 min for baseline; 11 min for follow-up). The average time for semi-automatic measurement of both baseline and follow-up diameters was 13 min (1 min for manual image preprocessing, 4 min for manual landmark annotation, 2 min for the processing of the baseline images, 6 min for the processing (registration and segmentation) and the automatic measurement of the follow-up images and the comparison with baseline).

## Discussion

Although the double-oblique technique has been regarded and used as the reference standard for aortic measurements, it is very time consuming and it has been associated with significant intra- and inter-observer variabilities (defined as mean difference ± SD) of − 0.8 ± 1.3 mm and 1.3 ± 2 mm, respectively and absolute difference values of up to 11 mm [13]. It has also been demonstrated, that the experience of the observers plays an important role in reducing the variability [14]. Therefore, notwithstanding standardization of the measurements, previous growth thresholds for intervention [2] have been removed and it is now suggested that only differences over time of more than 5 mm should be considered relevant [1].

Although several studies exist regarding the detection of the aorta on single time point CT scans using analytical software solutions [6–8, 15–17] only one previous study was published presenting a framework for semi-automatic/automatic comparison of baseline and follow-up abdominal aneurysms volume and diameters [4].

For single time point CT scans, several semi-automatic/automatic software packages for the thoracic aorta measurements have been validated and showed lower intra and inter-observer variability and reduced measurement time compared to manual measurements [6–8, 15–17].

The method developed by Martínez-Mera et al. [15] can segment the thoracic aorta completely automatically; the mean correlation coefficient of the aorta’s segmented volume of the 10 patients analyzed was 0.976. Vitanovski et al. [16] proposed a method that is able to detect the thoracic aorta and main branches automatically. The error was represented by the mean point-model Euclidean distance, which was found to be 2.29 ± 1.74 mm for the aorta. For Kovács et al. [17], the overall average bias of the patients was 1.1 ± 0.17 mm comparing the mean distance of the automatic and manual segmented aortic dissection meshes. In the study by Biesdorf et al. [6] the aortic arch was segmented by three different approaches: a model-based approach, a 2D joint approach and a 3D joint approach. The errors in the maximal diameters in the ten 3D CTA with mild pathologies of the aorta were 2.24 ± 0.72 mm, 1.51 ± 0.66 mm, and 1.52 ± 0.69 mm, respectively for the three methods. In the seven 3D CTA with severe pathologies, the errors were 5.45 ± 2.98 mm, 3.34 ± 2.23 mm, 2.04 ± 0.83 mm respectively. In Lu et al.’s study [7] the ascending aorta was semi-automatically measured by two observers. The inter-observer variability was 1.1 mm during the first session of measurements, and 1.2 mm during the second session. However, no comparison against manual reference standard was performed. The tool by Entezari et al. [8] which can segment the thoracic aorta semi-automatically, has the most similar features to our study; however, it only segmented and measured diameters without automatic comparison between two consecutive exams. The maximum diameters were measured manually and semi-automatically in multiple locations and the mean difference was calculated: all the differences were less than 1 mm, except at STJ and PROX locations.

In our study with the new tool, differences with baseline manual measurements were < 1 mm except at the STJ, MAA (only for one observer) and at the mid aortic arch, all ICC values > 0.90 and low inter-observer variability (< 1 mm differences with ICC values > 0.95 at all locations). Therefore, the contour detection algorithm for the baseline thoracic aorta has at least similar, or even better, accuracy and reproducibility compared to the published tools listed above.

The software [4] described by Kauffmann et al. relies on the semi-automatic segmentation of both datasets requiring the operators’ intervention at multiple steps, such as the user definition of the aortic lumen location and the correction of aortic contours. In Kauffmann’s study, the mean difference of the measured maximum cross-sectional diameters carried out by a senior radiologist and one of three medical students, was 0.07 mm and the ICC values for baseline and follow-up examinations were in the range from 0.989 to 0.998. In this study, the performance of the software was not compared to a reference standard, although in their previous study [5], they reported a mean error compared to manual measurement of 1.1 ± 0.9 mm.

To the best of our knowledge, no other software solutions have been described that can semi-automatically align the baseline and follow-up CT datasets of the thoracic aorta of the same patient, and allow the measurement of the diameters of both scans at the same time. In our study, the semi-automatic detection of changes in the follow-up thoracic aorta diameters showed differences < 1 mm compared to manual measurements, except at the STJ, mid aortic arch and MAA positions (for one observer). The inter-observer variability was < 1 mm at all locations, except at the MAA (1.04 mm), while the ICC were all higher than 0.95. The accuracy and inter-observer variability of the measurements on the follow-up scans of the thoracic aorta proved to be comparable to the results on baseline datasets. The measurement time was reduced compared to the manual measurements with the possibility of further improvements in the future.

Compared to the published studies, our software package presents the following new features: (1) the landmark locations can be identified automatically on the follow-up scan based on the baseline locations; (2) the contours of both the baseline and follow-up images can be compared automatically; and (3) the dilatation of the aorta (difference in diameters) between the baseline and the follow-up, can be visualized in color coding on a 3D reconstruction which gives an instantaneous overview of all relevant information.

There are some limitations in this study. First, the number of patients in this study is relatively small due to the strict inclusion criteria (patients with clinical reports presenting changes in aortic diameters; no congenital aortic anomalies or previous surgery; both baseline and follow-up scans with reasonable to high quality and thin slices reconstructions). Especially the choice to include only scans with reasonable to high quality does not reflect real world variability and further studies are needed to assess the quality of the software with a broader spectrum of qualities of the scans. In our study, only CT scans with not-acceptable image quality were excluded, which correspond to studies that are not diagnostic and that would therefore require further or repeated examinations for correct assessment of aortic diameters in clinical practise. Secondly, measurements were not compared to a true gold standard. However, this is a general issue for studies regarding aortic diameters assessment with imaging techniques. Even if the patients would undergo surgery at which time aortic dimensions could be derived, measurements performed in this setting cannot be considered the gold standard considering the limitations and difficulties of intraoperative measurements.

In conclusion, a novel semi-automatic tool which is able to align baseline and the follow-up images to allow the accurate measurement of the thoracic aorta at several landmarks, was developed and evaluated. This study has demonstrated that the tool has high accuracy and inter-observer variability, especially at PROX, DESC, and diaphragm locations. The processing time turned out to be decreased by 40% of manual measurements.

## Abbreviations

CT: Computed tomography
DESC: Mid descending aorta
DIST: Proximal descending thoracic aorta
MAA: Mid ascending aorta
MPR: Multiplanar reconstructions
PACS: Picture archiving and communications system
PROX: Proximal aortic arch
STJ: Sinotubular junction
